# The prairie vole gut–brain–microbiota-axis: a narrative review

**DOI:** 10.1017/gmb.2026.10021

**Published:** 2026-03-24

**Authors:** Daniel A. Nuccio, Angela Grippo, Pallavi Singh

**Affiliations:** 1 Department of Biological Sciences, Northern Illinois University, USA; 2 Department of Psychology, Northern Illinois University, USA

**Keywords:** gut–brain–microbiota axis, stress, metabolic diseases, prairie vole, metabolomics

## Abstract

The microbiota–gut–brain axis (MGBA) has garnered considerable attention for its role in health, disease, and higher psychological processes. One area of particular importance is the relationship between the MGBA and stress. Although numerous animal models are suitable for research on stress, the number suitable for research on the impact of social stressors with translatability to humans is limited. The prairie vole is regarded as an ideal organism for probing the impact of social stress, as these animals not only exhibit social behaviours rare in mammals but also lack many drawbacks that come with using non-human primates. Moreover, the neurophysiological basis of their social behaviours is well characterized, and numerous studies have examined the impact of social stress, particularly social isolation, on these animals. However, only a limited number of studies have examined the prairie vole gastrointestinal system, intestinal microbiome, or MGBA. Consequently, this leaves ample opportunity for future research. In this review article, we summarize basic aspects of prairie vole ecology, behaviour, and neurophysiology, then review the limited but valuable body of research examining the gastrointestinal system and microbiome of prairie voles. Additionally, we note potential challenges and opportunities for future MGBA research utilizing prairie voles.

## Introduction

The dynamic relationship between the nervous, endocrine, gastrointestinal, and immune systems, in addition to an organism’s microbiome, is increasingly gaining recognition for its role in health, disease, emotion, cognition, and behaviour. Often referred to as the microbiota–gut–brain axis (MGBA), the conceptual framework associated with this integrated system has been used to explore how gut microbial communities are involved in mental health and stress (Carabotti et al., 2015; Liu and Zhu, 2018; Ilchmann-Diounou and Menard, 2020; Misiak et al., 2020; Costa et al., 2024).

As a natural corollary, the impact of stress on gastrointestinal physiology has garnered attention, with studies showing that stress alters intestinal motility and permeability. Although the extent to which these changes are direct responses by the host to stress or downstream of changes in the host’s gut microbiome is unclear, these changes are of particular interest to researchers given their role in the development and progression of numerous pathologies. Increased intestinal permeability, whether caused by stress, perturbations to microbial communities, or diet, may lead to the passage of microbial endotoxins (e.g., lipopolysaccharide [LPS]) into the circulatory system, and low-grade systemic inflammation, in turn, resulting in cardiovascular and metabolic disease (Cani et al., 2007; Delzenne and Cani, 2011; Devaraj et al., 2013; Misiak et al., 2020; Wu et al., 2021; Zhang et al., 2021; Martel et al., 2022; Page et al., 2022; Salas-Venegas et al., 2022; Sadagopan et al., 2023; Costa et al., 2024). Additionally, the MGBA can influence an organism’s social behaviour and be influenced by its social environment. For instance, bacterial genera found in faecal samples from non-captive macaques can predict sociality, and the transplantation of the faecal microbiota of human patients with social anxiety disorder into mice can alter sensitivity to social fear (Johnson et al., 2022; Ritz et al., 2024).

However, despite the value of studying human clinical populations and samples from non-human primates, as well as performing experimental work using mice and rats, we present prairie voles (*Microtus ochrogaster*) as an underutilized model organism for unravelling the link between social stress and the MGBA. Unlike many other animal models, prairie voles share several social behaviours with humans that are generally considered rare in mammals. These include forming largely monogamous social pair bonds with mates, engaging in biparental care, and sometimes living in extended family units in which non-reproductive adult offspring remain in the parental nest and assist with the care of younger siblings (Carter and Getz, 1993; McGraw and Young, 2010; Kenkel et al., 2017; Kenkel et al., 2021). Furthermore, a large body of work amassed over the past 45 years provides a detailed characterization of prairie vole ecology and behaviour, the neuroendocrine basis of their social behaviours, and the effects of social stress on these animals (Carter and Getz, 1993; Grippo et al., 2007a, 2007b, 2007c, 2008; McGraw and Young, 2010; Kenkel et al., 2021; Normann et al., 2021).

Conversely, although mice and rats are utilized far more frequently in biomedical research, the social characteristics of these animals do not match those of humans as closely. Rats, although gregarious, do not form social pair bonds with mates, while the care of offspring is left solely to females. Mice, when living in natural or quasi-natural environments, sometimes form social systems considerably different than those of humans, as they can be characterized by extended family groups comprising a dominant male, multiple subordinate males, multiple reproductive females, and the offspring. Mice also do not form monogamous social pair bonds with familiar partners, although paternal care may show variability in lab settings based on mouse strain (Lonstein and De Vries, 2000; Lee and Beery, 2019). Additionally, although specific mutant strains (e.g., BTBR mice) may be useful models for understanding deficits in social behaviour associated with particular conditions, such as autism spectrum disorder, such animals can have genetic and physiological abnormalities that make them less suitable models for normal human social behaviours or how mammals with such behavioural profiles respond to social stress (Careaga et al. 2015).

Looking at future MGBA research, there is ample opportunity for the greater utilization of prairie voles in examining how social stressors act through the MGBA to influence gastrointestinal physiology and metabolic disease, as well as how gastrointestinal disturbances and gut microbiome changes can alter social behaviour. In the present review, we provide a detailed account of prairie vole ecology and behaviour, the neurophysiological basis of their social behaviours, and the impacts of social stress on these animals. We also discuss the small but valuable body of work that has already examined the prairie vole gastrointestinal system and gut microbiome, as well as potential limitations and opportunities when using these animals in MGBA research.

## Ecology and behaviour

Prairie voles are a species of rodent native to the central portions of North America (Roberts et al., 1998; Ophir et al., 2007). Despite being capable of reproducing year-round, wild prairie voles are less reproductively active during the winter (Cole and Batzli, 1979). Litters, which are born after ~22 days of gestation, typically contain three to six pups (Cole and Batzli, 1979; Solomon et al., 2004; Kenkel et al., 2017, 2023). Laboratory-bred prairie voles are commonly separated from their parents and housed with one or more same-sex siblings after 20–21 days to avoid exposure to subsequent litters, as this exposure can influence neurophysiology and behaviour (Lonstein and De Vries, 2000; Greenberg et al., 2012). In the wild, however, offspring often reside in the parental nest their entire lives, likely engaging in the care of younger siblings, while those that leave to start their own families may wait until they are 34–36 days old (Getz and Hofmann, 1986; Kenkel et al., 2017).

### Reproductive behaviour, pair-bonding, and biparental care

Females, although capable of reaching reproductive maturity and becoming sexually active at the time of weaning, require exposure to an unrelated, intact male or his soiled bedding to attain reproductive maturity (e.g., increased uterine weight and displays of behavioural oestrus in the form of lordosis; Carter et al., 1980, 1987; Witt et al., 1988). Mating in prairie voles occurs in extended bouts often lasting more than 24 hours (Witt et al., 1988). Unlike most mammals, after mating, prairie voles form long-term, quasi-monogamous social pair bonds, as well as engage in biparental care.

Although not all individuals are strictly sexually monogamous (Solomon et al., 2004), evidence of pair bond formation has been recorded both in the field and laboratory (Getz et al., 1981; Getz and Hofmann, 1986; Roberts et al., 1998; Barrett et al., 2013). Evidence of such relationships in the field comes from live-trap data of specific animals regularly captured near particular nests or repeatedly trapped with the same opposite-sex partner over time (Getz et al., 1981; Getz and Hofmann, 1986). In laboratory settings, pair bonding is operationally measured as a preference for spending time with a familiar opposite-sex partner with which an individual has mated or cohabited over a novel opposite-sex stranger, as well as increased aggression towards strangers of both sexes (Getz et al., 1981; Winslow et al., 1993; Roberts et al., 1998; Barrett et al., 2013).

Paternal care has been inferred from field observations of males residing in nests with partner females and their offspring, as well as recorded in laboratory settings (Getz and Hofmann, 1986; Roberts et al., 1998; Terleph et al., 2004; McGuire and Bemis, 2007; Ophir et al., 2007). Variability in paternal care has also been observed between populations. Male prairie voles from Kansas, for example, are less paternal than males from Illinois, potentially due to differences in responses to peripheral vasopressin, neural sensitivity to oestrogen, and their sometimes more xeric environments (Roberts et al., 1998; Cushing et al., 2001, 2004).

## Neurophysiology

Given the rarity of monogamous pair bonding and biparental care in mammals, the presence of such social characteristics in prairie voles has made them a preferred rodent model for research on the biological basis of these social behaviours. Consequently, for more than 30 years, researchers have used prairie voles to characterize the neurophysiological and endocrine basis of pair bonding and biparental care, as well as the impact of social isolation on social mammals. Investigations utilizing these animals have given considerable attention to oxytocin and vasopressin, although the roles of dopamine, corticotropin releasing factor (CRF), corticosterone (CORT), and opioid receptors have also been examined (Aragona et al., 2006; Barrett et al., 2013; Berendzen et al., 2023; Burkett et al., 2011; DeVries et al., 1995; Inoue et al., 2013; Keebaugh and Young, 2011; Lim et al., 2004; Lim et al., 2005; Lim et al., 2007; Liu and Wang, 2003; Resendez et al., 2012, 2013; Williams et al., 1994; Winslow et al., 1993).

### Oxytocin and vasopressin

Central oxytocin and vasopressin activity, often respectively in the female nucleus accumbens (NACC) and male ventral pallidum (VP), have long been associated with social behaviours in prairie voles (Winslow et al., 1993; Williams et al., 1994; Liu and Wang, 2003; Lim et al., 2004; Keebaugh and Young, 2011; Barrett et al., 2013; Berendzen et al., 2023). Research has demonstrated that infusions of oxytocin into the lateral ventricle of ovariectomized females induce a preference for a partner male over a novel stranger, while simultaneously infusing an oxytocin antagonist blocks partner preference formation (Williams et al., 1994; Liu and Wang, 2003). Furthermore, overexpression of oxytocin receptors (OTRs) in the female NACC also induces more affiliative behaviours towards partners by females and more alloparental behaviours towards unfamiliar pups (Keebaugh and Young, 2011). Notably, however, one recent study utilizing prairie voles lacking functional OTRs has called into question the necessity of oxytocin for pair bonding in prairie voles, as both males and females lacking OTRs still exhibited pair bonding (Berendzen et al., 2023). Similar studies of the role of vasopressin in social monogamy in male prairie voles demonstrate that blocking V1a receptors (V1aRs) in the brain before mating can prevent selective aggression towards unfamiliar conspecifics and partner preference formation, while downregulating V1aRs in the VP impairs partner preference formation without impacting alloparental behaviours (Winslow et al., 1993; Barrett et al., 2013).

### Dopamine, corticotropin releasing factor, corticosterone, and opioid receptors

Research into the roles of other potential neurochemical and endocrine mediators of social behaviour highlights several additional mechanisms, with many acting in the NACC and other parts of the striatum (e.g., caudate putamen [CP]). In the NACC, the binding of dopamine to D2-like receptors (D2Rs) facilitates pair bond formation while binding to D1-like receptors (D1Rs) prevents it (Liu and Wang, 2003; Aragona et al., 2006; Loth and Donaldson, 2021). CRF activity in the NACC, likely mediated through CRF1 and CRF2 receptors, helps facilitate partner preference formation in males (Lim et al., 2007). In females, *μ*-opioid receptors (MORs) in the rostral dorsomedial area of the NACC shell and CP help facilitate partner preference formation, while MORs in the dorsal striatum are important for mating behaviour (Burkett et al., 2011; Resendez et al., 2013). Additionally, *κ*-opioid receptors in the NACC shell play a role in selective aggression, although MORs in this region do not (Resendez et al., 2012). Peripherally, CORT, a stress hormone released downstream of the actions of CRF, blocks the formation of new pair bonds but does not effect established pair bonds when administered to female prairie voles (DeVries et al., 1995; Cacioppo et al., 2015a).

## Social isolation as a form of chronic stress

Mammalian stress responses can be activated by real or perceived threats, trauma, minor chronic stressors, psychosocial stress, and, in humans, maladaptive coping mechanisms. Such responses are largely mediated by the hypothalamic–pituitary–adrenal (HPA) axis and sympathetic nervous system (SNS;Carabotti et al., 2015; Cacioppo et al., 2015a; Misiak et al., 2020).

When the HPA axis is activated by signals from the prefrontal cortex (PFC), as well as limbic regions such as the amygdala and bed nucleus of the stria terminalis (BNST), CRF is released from the hypothalamus. CRF then acts on both the locus coeruleus and the anterior pituitary. The locus coeruleus, which can also receive signals from the PFC and various limbic structures, proceeds to release norepinephrine throughout the brain, while the anterior pituitary releases adrenocorticotropic hormone (ACTH) into the animal’s circulation, where it is transported to the adrenal glands. Upon activation by ACTH, the adrenal cortices release CORT into the animal’s blood, where it is transported throughout the body (Carabotti et al., 2015; Cacioppo et al., 2015a; Misiak et al., 2020). CORT’s major effects include increased gluconeogenesis, increased protein metabolism, and attenuation of immune responses (Misiak et al., 2020). Notably, CORT also serves as a negative feedback mechanism, acting on both the hypothalamus and anterior pituitary (Misiak et al., 2020). The SNS comprises numerous sympathetic nerve fibres that act on major organ systems both directly through the release of norepinephrine and indirectly through the stimulation of the adrenal medulla, which, in turn, releases epinephrine and norepinephrine, which then influence the functions of various organs, as well as metabolic and immune activity (Cacioppo et al., 2015a).

Although beneficial in the context of surviving an immediate or short-term threat, chronic activation of these systems, even when stressors are relatively mild, can lead to low levels of systemic inflammation and signs of metabolic disease (McEwen, 2007; Cacioppo et al., 2015a; Gjerstad et al., 2018; Misiak et al., 2020). Additionally, *in vitro*, norepinephrine is associated with the increased growth and possible virulence of some pathogenic bacterial species (Anderson and Armstrong, 2006; Cogan et al., 2007).

### Effects of social stress on social mammals

For social mammals, extended social isolation is a form of chronic stress that affects such organisms through the same systems as more standard stressors and has comparable effects on health and well-being (Hawkley et al., 2012; Cacioppo et al., 2015a). In humans, perceived loneliness is associated with premature death and reduced mental, cardiovascular, cognitive, and metabolic health (Cacioppo et al., 2015b; Valtorta et al., 2016; Holt-Lunstad, 2018; Sutin et al., 2020; Henriksen et al., 2023). Additionally, isolation following a stressful life event is associated with greater symptoms of clinical illness following rhinovirus exposure (Cohen et al., 2012).

Given the highly social nature of prairie voles and extensive research on the biological basis of their social behaviours, these animals have long been considered an excellent model organism for exploring questions about the impact of social stressors on social mammals in general and social isolation more specifically. In social isolation studies utilizing prairie voles, animals are generally housed either with a same-sex sibling or in isolation for 4–8 weeks, then behaviourally assessed before euthanasia and tissue sample collection (Grippo et al., 2007a, 2007b, 2007c, 2008; Normann et al., 2021).

Behavioural assessments typically entail placing animals in one or more behavioural assays to measure proxies of anxiety and depression. The elevated plus maze (EPM), forced swim test (FST), and sucrose preference test (SPT) are among those that have been utilized (Grippo et al., 2007a, 2007b, 2007c, 2008; Normann et al., 2021). The EPM is used to assess behavioural signs of anxiety (Walf and Frye, 2007; Normann et al., 2021). The FST and SPT are behavioural tests of depressive symptomology (Grippo et al., 2002, 2003, 2008; Strekalova et al., 2004). Sometimes, additional physiological measures (e.g., heart rate and heart rate variability) are also collected (Grippo et al., 2007c, 2008).

Analyses generally show isolated prairie voles engage in more behaviours indicative of anxiety and depression than paired controls (Grippo et al., 2007a, 2007b, 2007c, 2008; Normann et al., 2021). Additionally, isolated prairie voles demonstrate more signs of cardiovascular dysfunction, including tachycardia and decreased heart rate variability, likely due to dysregulated autonomic nervous system (ANS) activity (Grippo et al., 2007c, 2008). Research on social isolation’s cardiovascular and autonomic effects indicates isolated prairie voles physiologically respond to non-threatening environments in the same manner they respond to threatening ones, as well as take longer to recover from additional social stressors like being placed in the cage of an unfamiliar conspecific as part of a resident intruder test (RIT; Grippo et al., 2007c).

Studies examining the neurophysiological and hormonal effects of social isolation on prairie voles beyond the ANS, although sometimes inconsistent, generally reveal changes in parts of the brain and in central and peripheral neurohormones associated with both stress and social behaviour such as increased CRF immunoreactivity in the paraventricular nucleus of the hypothalamus (PVN) and vasopressin immunoreactivity in the supraoptic nucleus of the hypothalamus (Ruscio et al. 2007).

Some sex-specific differences in responses to social isolation have also been found in prairie voles. Females, but not males, show an increase in circulating OT, an increase in OT immunoreactive cell density in the PVN, and a decrease in AVP immunoreactivity in the PVN (Grippo et al., 2007b; Ruscio et al. 2007). Findings regarding the impact of social isolation on circulating CORT have been inconsistent (Donovan et al., 2020b; Grippo et al., 2007b; Ruscio et al., 2007).

Research assessing whether social isolation alters how prairie voles respond to additional social stressors, such as those experienced in the RIT, shows that following the RIT, isolated females exhibit elevated circulating oxytocin, vasopressin, and CORT, as well as more oxytocin and CRF immunoreactive cells in the PVN (Grippo et al., 2007a, 2007b). Circulating ACTH has sometimes been shown to be elevated in isolated females following the RIT (Grippo et al., 2007a, 2007b). The impact of social isolation on responses to other social stressors for males appears less pronounced, with isolated males showing increases in oxytocin immunoreactive cells in the PVN and circulating oxytocin following the RIT but no changes in CRF immunoreactive cells in the PVN or circulating ACTH or CORT (Grippo et al., 2007b).

Compared to other rodent models of stress (e.g., chronic unpredictable mild stress [CUMS]), the behavioural and physiological effects of social isolation on prairie voles are broadly consistent (Grippo et al., 2002, 2003; Walf and Frye, 2007; Jianguo et al., 2019).

## Prairie vole gastrointestinal physiology and gut microbiota

Despite the extensive body of work examining the nervous and endocrine systems of prairie voles, and the impact of social isolation on these systems, there has been limited examination of the prairie vole gastrointestinal system and microbiome, or how it is impacted by social isolation, leaving ample opportunity for further inquiry (Assefa et al., 2015; Curtis et al., 2018; Supeck et al. 2018; Donovan et al., 2020a, 2020b; Hume et al., 1993; Kenkel et al., 2023; Nuccio et al., 2023; Young Owl and Batzli, 1998). Animal microbiomes comprises the microbial organisms living in or on a host, although many discussions focus specifically on bacteria. In humans, estimates suggest bacterial cells roughly equal the number of host cells and include representatives from 500 to 1000 species, although this number ultimately may be substantially higher as previously uncharacterized taxa are discovered (Sender et al., 2016; Gilbert et al., 2018; Pasolli et al. 2019). Across mammalian microbiomes, Firmicutes and Bacteroidetes are the dominant bacterial phyla present, exhibiting considerable variability in species (Krych et al., 2013; Bleich and Fox, 2015; de Jonge et al., 2022). Evolutionary history, diet, and stress are among the factors that influence microbiome composition, as is exposure to natural environments (Gilbert et al., 2018; de Jonge et al., 2022). Generally, the microbiomes of wild animals are more diverse than those of their captive counterparts (de Jonge et al., 2022). In rodents specifically, studies comparing the gut microbiota of lab-raised animals and wild conspecifics demonstrate this pattern in mice (*Mus musculus domesticus*) and deer mice (*Peromyscus maniculatus*; Rosshart et al., 2019; Schmidt et al., 2019).

Different body sites and their subdivisions are generally treated as distinct ecosystems by microbiome researchers. Of the different body sites, the gut tends to be the most intensely studied, likely because the colon microbiome contains a greater number of bacterial cells and greater community diversity than other locations (Fukuda and Ohno, 2014; Bleich and Fox, 2015). The potential influence of these communities on host behaviour, physiology, and disease is among the dominant topics explored in microbiome research (Gilbert et al., 2018; Johnson and Foster, 2018; Wu et al., 2021). In part, this is likely because perturbations to an animal’s gut microbiome play a role in numerous diseases, including inflammatory bowel disease, colorectal cancer, diabetes, obesity, and Alzheimer’s (Delzenne and Cani, 2011; Devaraj et al., 2013; Kostic et al., 2014; Halfvarson et al., 2017; Zhang et al., 2021; Noureldein et al., 2022; Molinero et al., 2023; Sadagopan et al., 2023).

Although further investigation is necessary for the determination of precise mechanisms, evidence suggests certain microbes induce inflammation in the gut and damage gut barriers either directly or through components of their cell wall (e.g., LPS) and byproducts of their metabolism (e.g., ethanol); moreover, research shows once this damage occurs, LPS, whole bacteria, and various chemicals and antigens can enter host circulation and cause further inflammation or damage organs such as the liver and pancreas (Fukuda and Ohno, 2014). Conversely, there is also considerable research focused on the identification of probiotic bacterial taxa that can improve host health and alleviate disease (Fukuda and Ohno, 2014; Wolfe et al., 2023).

### Prairie vole gastrointestinal anatomy and physiology

Early research examining the prairie vole gastrointestinal system largely focused on basic anatomy and physiology, often approaching questions from an ecological perspective. This work elucidated how food intake by prairie voles appears to be limited by the amount of time necessary for digestion, thus requiring animals to engage in regular feeding bouts every 2–4 hours, interspersed with periods of rest (Zynel and Wunder, 2002).

Such studies also provided morphometric and functional details concerning different prairie vole gastrointestinal areas, revealing the caecum to have a greater nominal surface area compared to other hindgut regions and considerable acetate abundance in the caecum and proximal colon (Hume et al., 1993). This work also demonstrated proline uptake in the small intestine via an active transport system, greater acetate uptake in the distal colon than in the caecum or proximal colon through a passive mechanism when acetate is present in high concentrations, and equal levels of acetate uptake in the proximal and distal colon via a mediated system when acetate is present in low concentrations, as might occur during periods of fasting or starvation (Hume et al., 1993).

### Prairie vole gastrointestinal development

More recently, a limited number of studies have examined histological, cellular, and molecular aspects of the prairie vole gastrointestinal system. One of the most comprehensive studies, Supeck et al. (2018), provided a detailed description of the postnatal maturation of the intestinal epithelial barrier in prairie vole pups. Using animals ranging in age from 2 to 8 weeks, Supeck et al. (2018) characterized the development of the prairie vole intestinal epithelial barrier using a variety of techniques. Dextran permeability assays revealed greater intestinal permeability in 2-week-old animals compared to 3-week-old animals. Histological comparisons of colon tissue from 1- and 3-week-old animals demonstrated that 3-week-old animals had a greater number of goblet cells, increased smooth muscle width, and greater gland length and distance. Examination of the expression of genes involved in tight junction formation and paracellular permeability showed several such genes were upregulated at 3 weeks compared to 2 weeks. Subsequently, Supeck et al. (2018) concluded the prairie vole intestine is mature by 3 weeks of age, suggesting this would be the minimum appropriate age for using prairie voles in MGBA research, given that a mature intestinal epithelium would decrease permeability to macromolecules that could act as confounding factors.

Later work by Kenkel et al. (2023), examining the impact of caesarean delivery on prairie vole behaviour and MGBA physiology, found that adult male prairie voles delivered in this manner, when compared to animals delivered vaginally, exhibited a lower expression of multiple proteins relevant to gut maturation. This study also found that such animals exhibited lower abundances of the bacterial family *Prevotellaceae* and higher abundances of the taxa *Clostridiales* and *Ruminococcus.* No procedures were performed, however, to determine if these reported differences were causally linked or accompanied by abnormalities in gastrointestinal histology or functioning.

### *Prairie vole gut microbiome* c*haracterization*

Other investigations of the prairie vole microbiome include the assembly of five metagenome-assembled genomes from members of the prairie vole gut microbiome, a report on the general composition of the prairie vole gut microbiome, two studies examining the impacts of social isolation, and two studies assessing bacteria for potential probiotic properties or effects (Assefa et al., 2015; Curtis et al., 2018; Donovan et al., 2020a; Donovan et al., 2020b; Donovan et al., 2023; Nuccio et al., 2023). In addition to the study focused solely on the characterization of the prairie vole microbiome, such data are also reported in both social isolation studies and one of the probiotic studies (Curtis et al., 2018; Donovan et al., 2020b; Donovan et al., 2023; Nuccio et al., 2023).

Reports on the general composition of the prairie vole gut microbiome indicate Bacteroidetes and Firmicutes are the most abundant phyla, while *Muribaculaceae*, *Prevotellaceae*, *Ruminococcaceae*, and *Lachnospiraceae* are the most abundant families (Curtis et al., 2018; Donovan et al., 2020b; Donovan et al., 2023; Nuccio et al., 2023). These findings are consistent with descriptions of the gut communities of humans and other rodent models at the phylum level; they also bear some family-level similarities (Krych et al., 2013; Nagpal et al. 2018).

Notably, though, there have been some differences in the exact proportional abundances of some taxa between studies, as well as within studies. For example, the first study to characterize the prairie vole gut microbiome reported Firmicutes to make up 58.4% of gut communities and Bacteroidetes to make up only 26.96% of gut communities (Curtis et al., 2018). However, other researchers have found a greater proportion of the gut microbiota comprising Bacteroidetes, with some reporting this phylum to make up at least 50% of the prairie vole gut microbiome and Firmicutes to account for 40% or less of its composition (Donovan et al., 2020b, 2023). Additionally, one study that used two different sequencing techniques to process the same samples found inconsistencies in community composition (Donovan et al., 2023).

### The impact of social isolation on the prairie vole gut microbiome

The two studies that examined the impacts of isolation on the prairie vole gut microbiome both followed the basic experimental design of previous work in this area, but included faecal collection and analysis for microbiome comparison (Donovan et al., 2020b; Nuccio et al., 2023).

Donovan et al. (2020b) entailed the placement of prairie voles in either paired or isolated housing conditions for 6 weeks, with faecal collections gathered both before and after this period. Microbiome comparisons revealed isolated voles to exhibit a decrease in the abundance of an uncultured *Anaeroplasma* bacterium and an increase in the abundance of an uncultured *Candidatus Saccharimonas* rumen bacterium, an uncultured *Desulfovibrio* bacterium, and *Gastranaerophilales.* Paired animals exhibited a decrease in uncultured *C. Saccharimonas* rumen bacterium and an increase in a *Clostridiales* vadinBB60 group species. Additionally, sex-specific changes were observed, including a decrease in an uncultured *Ruminococcaceae* member in isolated females. Notably, members of the *Desulfovibrio* genus are broadly associated with disease, although some members may provide benefits to their hosts, while members of the *Ruminococcaceae* family are known for breaking down plant material from a host’s diet and the production of SCFAs, an important source of energy for colonocytes and a potential regulator of metabolic health (Flint et al., 2007; Wu et al., 2018; Zhou et al., 2024).

Other analyses indicated isolated animals showed increased behaviours associated with anxiety, a greater expression of CRF in the NACC, an increased expression of CRF and brain-derived neurotropic factor in the amygdala, and, in isolated males, increased circulating CORT.

The second, Nuccio et al. (2023), entailed the placement of female prairie voles in either paired or isolated housing for 4 weeks, with faecal collections gathered before the start of this period, followed by weekly collections for the next 4 weeks, as well as collection of faecal content directly from the colon. Emphasized findings included a greater abundance of *Anaerostipes* in paired animals and a greater abundance of *Staphylococcaceae* and *Enterococcaceae* in isolated animals when data from all faecal time points were pooled, as well as greater abundances of *Lactobacillaceae* in paired animals and *Vampirovibrio* in isolated animals when data from the final time point were considered alone. Notably, *Lactobacillaceae* and *Anaerostipes*, respectively, are associated with the regulation of pathogen growth and the greater production of SCFAs, while *Enterococcaceaeus* and *Staphylococcaceae* are families with prominent members associated with disease (Kant et al., 2015; Fiore et al., 2019; Chia et al., 2020; Bui et al., 2021; Zawistowska-Rojek et al., 2022).

This study also reported that isolated animals exhibited behavioural signs of both anxiety and depression. Furthermore, upon examining the faecal and serum metabolomes of their animals, the authors reported several metabolomic differences between paired and isolated animals that they argued were indicative of declining health in isolated animals. Specifically, they suggested that lower serum lactic acid and higher serum sorbitol and glyoxylic acid observed in isolated animals were consistent with the development of pre-diabetes or type-2 diabetes, while increased faecal succinate in isolated animals was consistent with an earlier characterization of colitis in a rodent model (Preston and Calle, 2010; Nikiforova et al., 2014; Osaka et al., 2017; Mora-Ortiz et al., 2019).

Despite the differences in specific bacterial taxa found by the two studies when isolated and paired animals were compared, both studies seemed to reveal isolated animals to have greater abundances of taxa associated with poor health and lower abundances of putatively beneficial taxa, including those associated with SCFA production. The metabolite profiles reported by Nuccio et al. (2023) also indicate that isolated animals were less healthy. However, neither study directly examined the physiological mechanisms that mediated the differences reported. They also did not directly examine whether the microbiome or metabolite profiles observed resulted in gastrointestinal or metabolic disease.

### Probiotics

Of the two published, peer-reviewed probiotic studies utilizing prairie voles, one focused on the identification of possible probiotic bacteria, while the other focused on the effects of administering what was thought to be a probiotic species (Assefa et al., 2015; Donovan et al., 2023).

The former characterized 30 *Lactobacillus* strains found in the prairie vole gut, assessing them for their probiotic potential (Assefa et al., 2015). Five were found to adhere well to intestinal epithelial cells, tolerate low pH environments and bile, and demonstrate anti-fungal and anti-bacterial properties, inhibiting the growth of *Candida albicans*, *Escherichia coli*, *Pseudomonas aeruginosa*, and *Staphylococcus aureus.*

The latter study examined how administering *Limosilactobacillus reuteri* to prairie voles impacted their behaviour, neurophysiology, and gut microbiota (Donovan et al., 2023). In this study, however, the researchers did not have a true control condition, instead comparing animals receiving live *Lactobacillus reuteri* to those receiving heat-killed *L. reuteri.* Interestingly, they found animals administered live bacteria exhibited less social behaviour, more CRF, and fewer vasopressin receptors in the PVN, and less CRF and fewer CRF2 receptors in the NACC, while males exhibited more CRF in the amygdala. Upon examining the possible impact of administering live or heat-killed *L. reuteri* on the gut microbiome, the researchers found numerous differences in taxa, including an increase in the potentially beneficial species, *Bifidobacterium boum*, in females administered heat-killed *L. reuteri* and decreased abundances of the potential pathogen, *Capnocytophaga canis.* Additionally, when performing correlational analyses on different neurophysiological measures and gut bacteria, they found in females positive correlations between *Methanobrevibacter oralis* and CRF in the PVN, *Prevotella* and CRF in the PVN, *Desulfovibrio* and vasopressin receptors in the PVN, and *Desulfovibrio* and both V1aRs in the PVN and CRF2 in the NACC.

The authors of this study attributed differences between animals receiving live and heat-killed *L. reuteri* to the administration of the bacterium, although, as with the social isolation studies, to incorporate a microbiome component, a precise mechanism was not identified through the work directly carried out in the study. However, the authors did note that the administration of heat-killed *Lactobacillus* species has been reported to yield physiological and behavioural effects on animals in previous studies, potentially through bacterial proteins, lipids, or metabolites that remain after bacteria are killed. They also suggested that the lower sociality exhibited by animals administered live *L. reuteri* was potentially linked to greater amounts of CRF in the PVN and deficits in CRF and CRF2 in the NACC in these animals, as the actions of CRF on CRF2 in the NACC previously have been linked to social behaviour in prairie voles. Additionally, it is worth noting that, given this study did not have a true control condition, it is unclear whether the administration of live *L. reuteri* led to some of the apparent deficits in these animals, had no effect, or perhaps improved animals compared to an unmeasured baseline but not as much as heat-killed *L. reuteri.*

### Microbiological research in prairie voles beyond the gut

Microbiological research utilizing prairie voles for investigations outside the gut has been limited. One study from the social isolation literature that examined the effects of social isolation and aggregate stressors on immune functioning using a bacterial killing assay found that plasma from males housed with same-sex siblings showed greater antibiotic capabilities against *E. coli* than plasma from isolated counterparts (Scotti et al., 2015). It also demonstrated plasma from isolated females to have less antibiotic capability following placement in the RIT than that of paired females, suggesting social stressors, including isolation, can have an aggregate effect in attenuating immune functioning (Scotti et al., 2015). A second study which, to the best of our knowledge, is the only published study to examine any component of the prairie vole microbiome outside of the gut and the only prairie vole microbiome study to use animals housed outside of a standard lab environment, characterized the oral microbiome of prairie voles in a lab setting, as well as that of animals transferred to semi-natural outdoor enclosures (Sabol et al., 2023). This study found Firmicutes to be the dominant phylum in the prairie vole oral microbiome, followed by Proteobacteria, Bacteroidetes, Actinobacteria, and Fusobacteria, regardless of whether animals were housed in a lab or an outdoor enclosure. At the family level, they established what they described as a core oral microbiome comprising 14 families. Upon comparing the oral microbiomes of prairie voles housed in the lab and in the outdoor enclosures, they found prairie voles exhibited greater alpha-diversity when in the outdoor enclosures and speculated this was due to differences in diet or oral contact with soil when burrowing or foraging. Changes in oral communities after animals were placed in outdoor enclosures entailed a decrease in the abundance of core families and an increase in others. Notably, the authors also reported that sociality had no influence on oral microbiome communities and that the oral microbiomes of siblings became more dissimilar as animals spent more time in the outdoor enclosures, suggesting the immediate environment may have a greater influence on the prairie vole oral microbiome than genetic relatedness.

## Considerations for future research

Previously proposed directions for future research regarding the prairie vole MGBA include investigating the MGBA’s potential role in social behaviours like mate choice and parental care, how this system is altered in paired or parental animals, whether isolation-induced gut microbiome perturbations can be reversed through probiotics, and whether isolation-induced gut microbiota changes broadly associated with metabolic disease are indicative of disease development in these animals (Assefa et al., 2015;Donovan et al., 2020b; Nuccio et al., 2023). Moving forward, certain considerations should be taken into account by researchers, although it should be noted that many of the matters to which these considerations pertain are not unique to the use of prairie voles.

### Procedural considerations in microbiome research

In microbiome research, there are numerous decisions researchers must make that can impact the results and interpretation of their work. These include how samples are collected and stored, the means by which bacterial cells are lysed, which hypervariable regions of the 16S rRNA gene are selected for amplification, which sequencing method is implemented, and how quality control measures are set before taxonomic assignment (Robinson et al., 2016; Gloor et al. 2017; Nearing et al., 2022).

Differences in such decisions regarding protocol may, in fact, account for some of the discrepancies in the composition of the prairie vole faecal microbiome seen across studies in which the general compositions reported were similar, but specific percentages for the proportional abundance of even major taxa were quite different (Curtis et al., 2018; Donovan et al., 2020b, 2023; Nuccio et al., 2023). Such considerations were also highlighted in one prairie vole microbiome study in which multiple sequencing techniques were used on the same samples, but yielded different results (Donovan et al., 2023).

Similarly, the tools and analyses used for statistical comparison can influence one’s results, as such tools and analyses may differ in their false discovery rates and their compatibility with the compositional nature of microbiome datasets (Gloor et al. 2017; Nearing et al., 2022). Potentially, differences such as tools and analyses may help explain the inconsistencies in findings from different microbiome studies, such as those of the two social isolation studies that examined the prairie vole microbiome (Donovan et al., 2020b; Nuccio et al., 2023).

### Animal models

Despite the noted value of animal models in biomedical research, there is growing concern over conflicting results across studies using animal models and the implications this can have for translating findings from lab animals to their wild counterparts or to humans in clinical settings (Bleich and Fox, 2015; Omary et al., 2016; Rosshart et al., 2019). Suggested remedies include stricter and more standardized reporting requirements for variables like age, sex, genetic background, bedding composition, and diet (Omary et al., 2016).

Of the variables enumerated above, diet is of particular importance for MGBA research, as it can impact gut anatomy, physiology, and microbiome composition, as well as behaviour, cognition, and metabolic health (Pellizzon and Ricci, 2020; Thigpen et al. 2004). However, despite its importance, the authors of relevant reviews highlight that information regarding diet composition, nutrient concentration, and the possible inclusions of non-nutritive components (e.g., phytoestrogens, heavy metals, mycotoxins, and endotoxins), to the extent such details can be known, are generally omitted from published research reports. Moreover, the authors of such reviews note how even commonly used laboratory diets purchased from manufacturers can vary considerably and affect animal phenotype (Pellizzon and Ricci, 2020). Specifically, such authors note how grain-based diets can vary in the concentration of the ingredients, nutrients, and non-nutritive components present, while purified diets, although more consistent and less prone to contamination, may be deficient in the kinds of fibres used by gut microbes for fermentation (Pellizzon and Ricci, 2020).

Additionally, for work examining the microbiome, researchers have highlighted that free-living animals, including humans, evolved in a “microbial world,” and that those living in “sanitized environments” are ecologically and microbially different (Rosshart et al., 2019). This has been repeatedly demonstrated, including in studies examining the microbiomes of different rodents (Rosshart et al., 2019; Schmidt et al., 2019; Sabol et al., 2023). It has also been suggested that failure to take this into account can have devastating results for human participants in clinical research, in part, due to differences in the immune functioning of humans and standard lab mice that are downstream of the latter having spent their entire lives in sanitized environments (Rosshart et al., 2019).

### Suitability of prairie voles for MGBA research and comparison to other models

The extent to which different concerns about the use of more standard lab animals, such as mice, apply to the use of prairie voles is largely unknown, given the dearth of research on this topic. Animal age and sex are routinely reported in prairie vole research, while possible sex differences are regularly discussed, if not examined directly. Genetically, animals are usually reported as being descended from wild prairie voles initially captured in central Illinois and regularly outbred to maintain genetic diversity within colonies.

#### Possible retention of wild features

Given the findings of research using “wilding” mice, the fact that prairie voles are regularly outbred may lend additional support for their use in MGBA and microbiome research. Wilding mice are created via the transfer of embryos from lab mice to wild mice. They can possess the genetic background desired by a researcher, but retain a more natural and less easily perturbed microbiome, as well as more natural immune gene expression (Rosshart et al., 2019).

Subsequently, we argue, it is reasonable to hypothesize that prairie voles retain more “wild” gut microbiomes and immune gene expression profiles. If this holds true, standard practices in prairie vole husbandry may facilitate the utilization of animals with more natural microbiomes and immune gene expression under controlled conditions.

However, we also acknowledge work with deer mice in which wild individuals were transferred to a lab setting has shown that the gut microbiomes of these animals become more similar to those of lab-born animals over time (Schmidt et al., 2019), suggesting prairie voles, once brought into the lab, may lose the features of a “wild” gut microbiome due to changes in diet, environment, and stress.

#### Prairie vole diet

Research pertaining to the effects of diet on prairie voles is limited. Early work on this topic demonstrated that prairie voles prefer dicotyledons such as alfalfa and clover to monocotyledons of comparatively lower nutritional value, but will still eat monocotyledons, and sometimes supplement their diet with seeds and insects, depending on what food items are available (Cole and Batzli, 1979). Prairie voles with access to preferred higher quality food exhibit greater life expectancy and reproductive success than conspecifics in habitats dominated by lower quality food items (Cole and Batzli, 1979). When fed more fibrous, less digestible grass instead of alfalfa, gastrointestinal changes occur (e.g., increased gastrointestinal tract size), presumably as a compensatory mechanism (Young Owl and Batzli, 1998). Additionally, when fed rabbit chow, they exhibit greater reproductive success and greater weight after weaning compared to animals fed a more natural diet of alfalfa (Cole and Batzli, 1979). Given that rabbit chows are routinely fed to prairie voles used in laboratory environments, this may suggest the baseline health of prairie voles used in research might be better than that of their wild counterparts. However, to the best of our knowledge, the physiological reasons and broader implications of these findings remain unexplored, as does the effect of diet and lab-housing on the prairie vole gut microbiome.

#### Comparison with other rodent models

As for the suitability of prairie voles as subjects for MGBA and microbiome research compared to other rodent models, we hold that these animals are a viable option for MGBA or microbiome research with a social component intended to have relevance to humans, most notably due to similarities in their social systems and behaviours (Lee and Beery, 2019). We also suggest that the applicability of prairie vole microbiome research to humans is likely comparable to the applicability of microbiome research utilizing other rodent models. Although direct comparisons of the human microbiome to those of various models are limited, such research suggests the microbiomes of humans and lab-housed mammals, including mice and rats, qualitatively share a large number of taxa, but that the proportional abundances of these taxa vary considerably (Krych et al., 2013; Nagpal et al. 2018). This has been suggested to likely be due to the evolutionary distance between species and differences in diet (Krych et al., 2013; Nagpal et al. 2018).

Although, to the best of our knowledge, direct comparisons of the human and prairie vole microbiomes have not been performed, previous descriptions of the prairie vole microbiome indicate it is reasonable to hypothesize similar patterns would emerge if direct comparisons were made (Curtis et al., 2018; Donovan et al., 2020b, 2023; Nuccio et al., 2023).

With that stated, we also acknowledge there are certain drawbacks to the use of prairie vole in MGBA and microbiome research. Much more is known about the MGBAs and microbiomes of mice and rats, which can make the development of future research questions and interpretation of subsequent results easier. Also, more is known about how the gastrointestinal systems of mice and rats compare to those of humans. Furthermore, the genetic and environmental distance of such animals from their wild counterparts helps reduce genetic variability, as well as microbial variability. However, once more, this can introduce different questions regarding how applicable one’s results are outside of a lab or beyond a specific strain of lab rodent (Rosshart et al., 2019).

### Future opportunities

Moving forward, there are ample opportunities for further exploring both basic and applied questions pertaining to the prairie vole MGBA. At present, it is unknown if and how the prairie vole gut microbiome varies between subpopulations or changes in females based on reproductive factors. Additionally, further investigation is required to determine the extent to which the prairie vole microbiome differs between wild and lab-housed animals, as well as the effect of such potential differences on prairie vole immune functioning.

As discussed, prairie voles offer relatively unique opportunities for researchers looking to examine social components of the MGBA. The utilization of the standard design implemented in social isolation studies in conjunction with measures of gut permeability offers researchers the opportunity to examine social stress’s effects on gut integrity (Donovan et al., 2020b; Nuccio et al., 2023). By also measuring the presence of inflammatory markers such as LPS and proinflammatory molecules, researchers can examine whether social isolation is associated with systemic inflammation, as has been done in the CUMS literature (Zhang et al., 2023). Additionally, given that elevated LPS in circulation and systemic inflammation are associated with poor metabolic health, one could test for indicators of declining metabolic health associated with LPS, such as increased fasted glycaemia and insulinaemia (Cani et al., 2007). One could also test whether the administration of specific probiotics can attenuate those or other negative health effects spurred by social isolation.

## Conclusion

The highly social nature of prairie voles, along with the large bodies of work concerning the biological basis of their social behaviours and the effects of social isolation on these animals, makes prairie voles ideal model organisms for investigating how the MGBA influences the social behaviour of social mammals, as well as how it is influenced by the social environment. Moreover, given that social isolation generally is viewed as a form of chronic stress for social mammals, and that chronic stress has been linked to gut microbiome perturbations, signs of increased gut permeability, and indicators of metabolic disease and systemic inflammation in other rodent species (Zhang et al., 2023), we argue that prairie voles could serve as an excellent rodent model for the exploration of how the stress of social isolation may act through the MGBA to impact metabolic health. However, given that the prairie vole microbiome is not as well characterized as that of other rodent models, further research is needed to establish what approximates a baseline or core prairie vole microbiome. Furthermore, researchers will need to explore how the prairie vole microbiome may differ between wild and lab-housed animals, and what implications this has on immune functioning when translating some basic research findings to clinical settings. They will also have to determine how factors such as subpopulation, sex, age, and diet may influence both the MGBA and gut microbiome. Yet, it is worth pointing out that these problems are not unique to those working with prairie voles, as there are comparable if not identical challenges researchers are still working to overcome, even when using more common animal models such as mice.
