# The prairie vole gut–brain–microbiota-axis: a narrative review – CORRIGENDUM

**DOI:** 10.1017/gmb.2026.10029

**Published:** 2026-06-15

**Authors:** Daniel A. Nuccio, Angela Grippo, Pallavi Singh

The authors regret the inclusion of two errors in the above article.

The first error is a spelling mistake in the third paragraph of the section “The impact of social isolation on the prairie vole gut microbiome”. “Enterococcaceae” is misspelled as “Enterococcaceaeus”.

The second error is in the second sentence in the third paragraph of the “Probiotics” section. “Lactobacillus reuteri” should read “L. reuteri”.

The authors apologise for these errors.
